# Ralph Ellison's Opportunity
†"Ralph Ellison's Opportunity; and East and West." By Leslie Keith. (The Religious Tract Society.)


**Published:** 1890-02-08

**Authors:** 


### Ralph Ellison's Opportunity.†

This volume contains two stories of which the first, "East and West," is the longer and better. Mr. Keith has a vigorous touch, and considerable skill in delineation of character. In "East and West" he has chosen the somewhat well-worn theme of a young poet's struggles in the asphyxiating atmosphere of Bethnal Green, but he has kept his individuality, despite a certain tendency towards Besant's treatment and style. Alicia Frankland, the heroine, is excellent, original, amusing, and womanly. The dialogue is crisp, and the story well put together. "Ralph Ellison's Opportunity" does not show quite so much ability perhaps, but it is wholesome and thoroughly readable, and the volume, which is well got up and contains several very fair illustrations, will form a sensible present for boys and girls alike.

